# Interaction of Amyloid Inhibitor Proteins with Amyloid Beta Peptides: Insight from Molecular Dynamics Simulations

**DOI:** 10.1371/journal.pone.0113041

**Published:** 2014-11-25

**Authors:** Payel Das, Seung-gu Kang, Sally Temple, Georges Belfort

**Affiliations:** 1 Computational Biology Center, IBM Thomas J. Watson Research Center, 1101 Kitchawan Road, Yorktown Heights, New York 10598, United States of America; 2 Neural Stem Cell Institute, Rensselaer, New York 12144, United States of America; 3 Howard P. Isermann Department of Chemical and Biological Engineering and Center for Biotechnology and Interdisciplinary Studies, Rensselaer Polytechnic Institute, Troy, New York 12180, United States of America; Weizmann Institute of Science, Israel

## Abstract

Knowledge of the detailed mechanism by which proteins such as human αB- crystallin and human lysozyme inhibit amyloid beta (Aβ) peptide aggregation is crucial for designing treatment for Alzheimer's disease. Thus, unconstrained, atomistic molecular dynamics simulations in explicit solvent have been performed to characterize the Aβ_17–42_ assembly in presence of the αB-crystallin core domain and of lysozyme. Simulations reveal that both inhibitor proteins compete with inter-peptide interaction by binding to the peptides during the early stage of aggregation, which is consistent with their inhibitory action reported in experiments. However, the Aβ binding dynamics appear different for each inhibitor. The binding between crystallin and the peptide monomer, dominated by electrostatics, is relatively weak and transient due to the heterogeneous amino acid distribution of the inhibitor surface. The crystallin-bound Aβ oligomers are relatively long-lived, as they form more extensive contact surface with the inhibitor protein. In contrast, a high local density of arginines from lysozyme allows strong binding with Aβ peptide monomers, resulting in stable complexes. Our findings not only illustrate, in atomic detail, how the amyloid inhibitory mechanism of human αB-crystallin, a natural chaperone, is different from that of human lysozyme, but also may aid *de novo* design of amyloid inhibitors.

## Introduction

Alzheimer's disease (AD) is the most common form of dementia affecting nearly 38 million people worldwide. The pathological hallmarks of AD are the aberrant deposition of extracellular senile plaques comprised of amyloid-beta (Aβ) peptides and intracellular neurofibrillary tangles [1]. Aβ isoforms of different lengths (ranging from 38 to 43) are generated by sequential cleavage of the amyloid precursor protein (APP) via proteolytic processing. Aβ40 and Aβ42 are the major isoforms generated via the “amyloidogenic” pathway by β- and γ-secretase. In addition, the Aβ_17–40/42_ fragments known as the p3 peptides are generated via “non-amyloidogenic” pathway by α- and γ-secretase.

The abnormal aggregation of the Aβ peptides into β-sheet rich fibrils involves a heterogeneous ensemble of oligomeric intermediates, all of which are found to be neurotoxic [2]. Aβ toxicity likely originates from a number of factors, including formation of ion channels [3], oxidative stress [4], interaction with receptors [5]. A recent study reported that the p3 (17–42) peptides undergo faster aggregation *in vitro* compared with Aβ_1–42_ peptides, while the fibril morphology and the oligomerization remain unaltered [6]. NMR data for the Aβ fibril structure proposed either parallel or anti-parallel orientations of the β-sheets [7]. Additional NMR studies [8]–[11] suggested Aβ_1–42_ fibril models as parallel-stacked hairpin-like structures of Aβ peptides. Residues 1–9/17 appear unstructured, whereas residues 18–42 form a β-strand–turn–β-strand hairpin motif that comprises two intermolecular, parallel, in-register β-sheets formed by residues 18–26 and 31–42.

**Figure 1:**
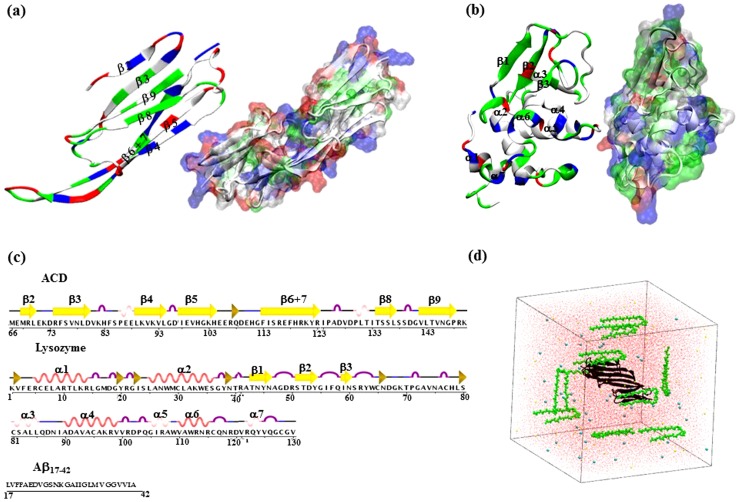
Structure and sequence of the simulated proteins. (a) Structure of an ACD monomer in cartoon and of the ACD dimer in surface representation colored according to residue type. Color scheme used: red - acidic, blue - basic, green - polar, and white - hydrophobic. (b) Cartoon and surface representations of the human lysozyme protein colored according to the residue type. (c) Sequence and the secondary structure assignment of human α-crystallin domain (ACD, residues 66–150) and of human lysozyme using the secondary structure assignment program DSSP [97]. Yellow arrows indicate β-strands, purple indicates turn, blue indicates bend, red spirals indicate the alpha- helix, light pink spirals indicate the 3_10_ helix, and black indicates coils. Sequence of the Aβ_17–42_ peptide is also shown. (d) System set up with one ACD dimer (black cartoon) placed in the center of a cubic box of water (shown in red) that also contains 10 Aβ_17–42_ peptides (green spheres, only backbone is shown). Sodium and Chloride ions are shown as cyan and yellow spheres, respectively.

Meanwhile, other proteins such as small heat shock proteins (sHsps) are also found co-localized with Aβ peptides in the amyloid plaque [12]–[14]. One of sHsps, αB-crystallin, has been extensively studied. αB-crystallin acts as an archetypical and ubiquitous ATP-independent molecular chaperone that binds partially unfolded polypeptides and maintains them in a refolding-competent state [15]–[17]. αB-crystallin is present in many parts of the human body including skeletal muscles and heart, and is a crucial component of the eye lens [18]. The native monomer of the αB-crystallin (∼175 residues) comprises a ∼90 residue β-sandwich domain that is termed the α-crystallin domain (ACD) and is highly conserved among all sHsps [19] (**Fig. 1a & 1c**). ACD is flanked by a variable, largely unstructured N-terminal region and a moderately conserved C-terminal extension [20]. It forms stable dimers that further assemble into a heterogeneous mixture of larger homo-oligomers [21]. Experiments suggest that ACD possesses considerable chaperone activity as well as contains interactive sequences against substrate proteins [22]–[25].

αB-crystallin is up-regulated in the brain of AD patients [13] and is also associated with Aβ deposition in the supranuclear cataract of the lenses of AD patients [26]. The fact that αB-crystallin is found co-localized with Aβ *in vivo* has stimulated *in vitro* experiments [27]–[34] to understand the effect αB-crystallin on Aβ aggregation. A majority of these experiments suggest that αB-crystallin can inhibit Aβ amyloid fibril formation by direct binding [30]–[32] and has an inhibitory effect on Aβ-associated toxicity and aggregation [29], [30], [35], [36]. Dobson and coworkers have found that αB-crystallin tightly binds with Aβ oligomers, thereby inhibiting their conversion to fibrils as well as their interaction with membrane [33], [34], which might help modulating the Aβ-mediated toxicity [37].

Interestingly, several other proteins that are not molecular chaperone have been reported to retard Aβ fibril formation as well [38]. For example, human lysozyme has been shown to inhibit Aβ aggregation *in vitro* at higher stoichiometric ratios than αB-crystallin [39]. The sequence, structure and the protein surface amino acid composition distinctively differ between lysozyme and ACD. Lysozyme is a primarily helical protein with a net +8 charge at pH 7 (see **Fig. 1b & 1c**). On the other hand, ACD shows a β-sandwich fold and has net charge of -2 (**Fig. 1a**). At present, limited data on the interaction between Aβ peptides and these amyloid inhibitor proteins exists, which is needed for designing novel protein therapeutics for AD.

Since most experimental approaches do not have the necessary resolution to determine inhibitor-peptide binding interactions at an atomic level, molecular dynamics (MD) simulations provide an alternative approach for such problem. MD simulations have been widely used to complement experiments [40] in providing detailed information on the structure of various Aβ species ranging from monomers [41]–[45] to oligomers [46], [47] to protofibrils [48] to fibrils [49]. Interactions of different Aβ species with toxicity and aggregation inhibitors [50]–[52] and with lipid bilayers [53]–[55] have been also studied using MD.

In the present study, unconstrained, atomistic MD simulations in explicit water are employed to characterize the effect of the structured core domain of αB-crystallin as well as of lysozyme on the assembly of Aβ_17–42_ peptides and the interaction between them (**Fig. 1d**). The choice of the Aβ17–42 fragment is motivated by several reasons: (1) the naturally occurring “amyloidogenic” 17–42 fragments of Aβ, known as the p3 peptides, which are constituents of AD amyloid plaques [56] and cerebellar pre-amyloid lesions in Down's syndrome [57], induce neuronal toxicity characteristic of AD [58], and form ion channels [59]. Recently, a crystal structure of the p3 fragment has been reported, providing a model for non-fibrillar Aβ oligomers [60]. (2) Those N-terminally truncated peptides are found to aggregate profoundly in *in vitro* experiments [6]. (3) The 17–42 fragment is comprised of the two hydrophobic patches (L17-A21 and A30–A42) and the turn region (E22-G29) that are crucial for determining aggregation and toxicity, form the strand-loop-strand conformation in fibrils, and also contain the vast majority of disease-causing mutations. (4) Aβ fragments have been the subject of many MD simulations in explicit water to offer useful atomistic information [42], [61], [62] [51], [63]. Finally, the N-terminal truncation lowers the computational expense significantly and allows us to obtain reliable statistics. Our simulations reveal distinct preference of individual inhibitor protein for binding to peptide monomers *vs.* oligomers. The molecular factors underlying such differences in the Aβ binding dynamics are further illustrated in detail.

## Results

### Effect of ACD and lysozyme binding on Aβ assembly

To study in detail the effect of the structured core domain (ACD) of a human αB-crystallin dimer and of a human lysozyme molecule on the Aβ_17–42_ assembly, we performed an aggregate of ∼4.5 µs of unconstrained, explicit solvent, atomistic MD. Due to limited available information on the putative Aβ binding modes with ACD and with lysozyme, brute force MD was used. We placed ten peptides randomly in the simulation box to explore multiple binding sites on inhibitors and their preference for peptide monomers *vs.* oligomers. A much higher Aβ concentration compared to experiments was used to expedite the association kinetics in simulations, while keeping the inhibitor:peptide ratio at 1∶10, which is close to experiments (see Model and Method section). Three systems were simulated: (i) ten peptides as a control system, (ii) ten peptides and one ACD dimer, and (iii) ten peptides + one lysozyme molecule. At least five different ≧200 ns runs were performed for each system at 325 K and 1 atm.

**Figure 2:**
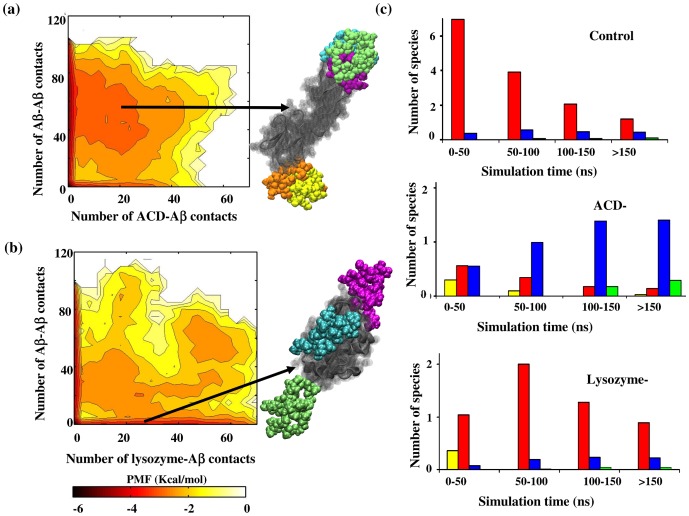
Effect of amyloid inhibitor proteins on Aβ assembly. 2D potential of mean force PMF plots as a function of the number of contacts between a peptide and the inhibitor protein (x axis) and the number of inter-peptide contacts for a peptide (y axis): (a) ACD and (b) lysozyme. Each contour level represents 0.5 kcal/mol free energy difference. The color scale for the free energy (kcal/mol) is shown at the bottom. Presence of ACD-bound oligomers is apparent. A snapshot of a crystallin-bound oligomer is shown, in which one Aβ dimer (yellow and orange) is attached to one α-crystallin domain and one Aβ trimer (green, cyan, and violet) is complexed with another α-crystallin domain. Crystallin domains are shown in gray. In contrast, lysozyme-bound monomers are primarily populated. A snapshot of lysozyme (in gray) is shown, in which three Aβ monomers (green, cyan, and violet) are found complexed. (c) Evolution of number of different sized Aβ species averaged over at least five different runs for each system. (a): control system (no inhibitor); (b): ACD-bound peptides; (c): lysozyme-bound peptides. Data is averaged over every 50 ns. Colored bars represent: yellow: uncomplexed ACD or lysozyme; red: Aβ monomer; blue: small Aβ oligomer (n = 2–5); green: larger Aβ oligomer (n>5).

To probe the competition between the peptide-peptide and peptide-inhibitor binding during the very early-stage (∼200 ns) of aggregation, we computed the two-dimensional potential of mean force plots as a function of (i) the number of contacts between the inhibitor and a peptide, and (ii) the number of contacts between a peptide and all other peptides (**Fig. 2**). ACD-complexed Aβ oligomers appear prevalent, as indicated by the higher probability of the configurations containing significant number of ACD-Aβ and inter-peptide contacts (**Fig. 2a**). A minor (1%) population of ACD-bound monomers is also noticed, as represented by the region on the PMF plot with number of ACD- Aβ number of contacts being ≥5 and number of inter-peptide contacts being <5. In contrast, lysozyme preferentially binds to peptide monomers, suggested by the dominant population of lysozyme-bound peptides that form limited contacts with other peptides (**Fig. 2b**). However, a smaller (4%) population with strong lysozyme-peptide binding (number of lysozyme-Aβ contacts>40) is also noticed, which also interact with other peptides (number of Aβ-Aβ contacts>40). Such population is not observed in the ACD case (see below).

These results are further confirmed by computing average number of different sized inhibitor-bound Aβ species at different time interval and comparing that with the results obtained for the control system (**Fig. 2c**). Results for all individual runs are found in the supplementary information (**Fig. S1–S3** in File S1). We considered monomers, small oligomers (n = 2–5) and larger oligomers (n>5) separately in this analysis. **Figure. 2c** reveals that the monomers and small oligomers are the prevalent species during the ∼200 ns simulation of control system (top panel). In presence of ACD, during the first 50 ns the number of complexed monomers and complexed small oligomers appear nearly the same (**Fig. 2c** **, middle**). As the simulation progresses, the number of ACD-bound monomer becomes lower with increasing presence of ACD-complexed small oligomers. The complexed oligomers remain present during rest of the simulation. Some population of larger complexed oligomers is also observed after 100 ns.

In contrast, peptide monomers dominate in lysozyme binding, as the monomers appear to be the prevalent complexed species (**Fig. 2c** **, bottom**). The population of small complexed oligomers remains constantly smaller than complexed monomers in presence of lysozyme. **Figure 2c** further indicates that the number of lysozyme-complexed monomers is higher compared with the ACD-bound ones during the total time-course of simulation. These results suggest that both inhibitors compete with the inter-peptide interaction by binding to the peptides. Such complexation would lower the effective concentration of the free peptides in solution, contributing to aggregation inhibition, which matches closely with experiments [31], [35], [36], [39]. However, the inhibition mechanism varies depending on the presence of ACD or lysozyme. For the ACD case, sequestration of the free small oligomers is observed, while monomers are primarily sequestered in presence of lysozyme.

### ACD-Aβ contact surface is less extensive

**Figure 3:**
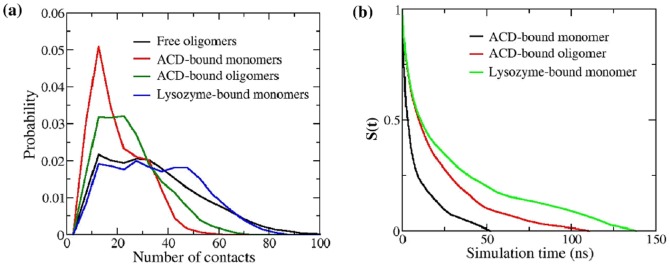
Binding and lifetime of inhibitor-peptide complexes. (a) Probability distributions of the number of heavy atom contacts formed between two Aβ peptides (black) in the control system (no inhibitor), between ACD and an Aβ monomer (red), between ACD and an Aβ oligomer (green), and between lysozyme and an Aβ monomer (blue). Lysozyme-bound Aβ oligomers were excluded from the analysis due to their minor population. At least five different ∼200 ns runs were used, in which multiple binding/unbinding events were observed. (b) Mean survival time correlation function, S(t), of peptides in the vicinity of the inhibitor: ACD-bound monomer (black), ACD-bound oligomer (red) and lysozyme-bound monomer (green). Each curve shows average of five independent runs. S(t = 0) measures the average number of peptide molecules bound with inhibitor, and S(t) gives the average number of peptide molecules that remain bound after a period of time, t, given that they were present at t = 0. A short escape of 1 ns was allowed during the calculation.

To compare the inhibitor-peptide and inter-peptide interaction, we computed the probability distribution of the number of contacts for the inhibitor-complexed monomers (ACD and lysozyme), complexed oligomers (ACD-bound only), and free oligomers (**Fig. 3a**). The complexes that have more than five heavy atom contacts were only considered for this analysis. The ACD-peptide monomer interaction appears weakest, as suggested by the peak location of the distribution. When a peptide from an Aβ oligomer binds to ACD, the ACD-Aβ interaction surface appears relatively more extensive compared to the scenario of a single monomeric peptide bound to ACD (indicated by the higher probability of forming more than ten contacts). In contrast, lysozyme-peptide binding appears nearly comparable to the inter-peptide binding in terms of the number of contacts (**Fig. 3a**). Consistently, the rate of decrease of the time-dependent survival probability shows the following order: ACD-bound monomer <ACD-bound oligomer <lysozyme-bound monomer (**Fig. 3b**). The evolutions of the number of complexed peptide monomers and oligomers during a typical trajectory show similar trend (**Fig. S4** in File S1). ACD first encounters with an Aβ monomer (t<25 ns), which dissociates shortly (**Fig. S5a** in File S1). In the later stage (t = ∼50 ns), one Aβ dimer and one Aβ trimer form complexes with ACD (**Fig. S4a** in File S1). Once formed, these ACD-bound small Aβ oligomers (one dimer and one trimer) remain attached for ∼100 ns. In contrast, during a typical trajectory amyloid monomers bind first with lysozyme (**Fig. S4b** in File S1). Over time, these complexed monomers can assemble to form complexed higher order aggregates (populating the upper right region of **Fig. 2b**). Taken together, these results suggest that the Aβ monomers make limited contacts with ACD, resulting in transient complex formation. The small oligomers, however, can form relatively more stable complexes with ACD by making higher number of contacts. The more stable interaction of an Aβ oligomer with ACD is due to the simultaneous contact formation of more than one monomer. It is also possible that the slower reconfiguration time [64] of the oligomer (compared with the monomer) allows more extensive interaction with ACD. In contrast, lysozyme interacts strongly with monomeric peptides, which is comparable to the inter-peptide surface, resulting in longer-lived complexes.

### Specific binding interactions of Aβ peptides with inhibitors

**Figure 4:**
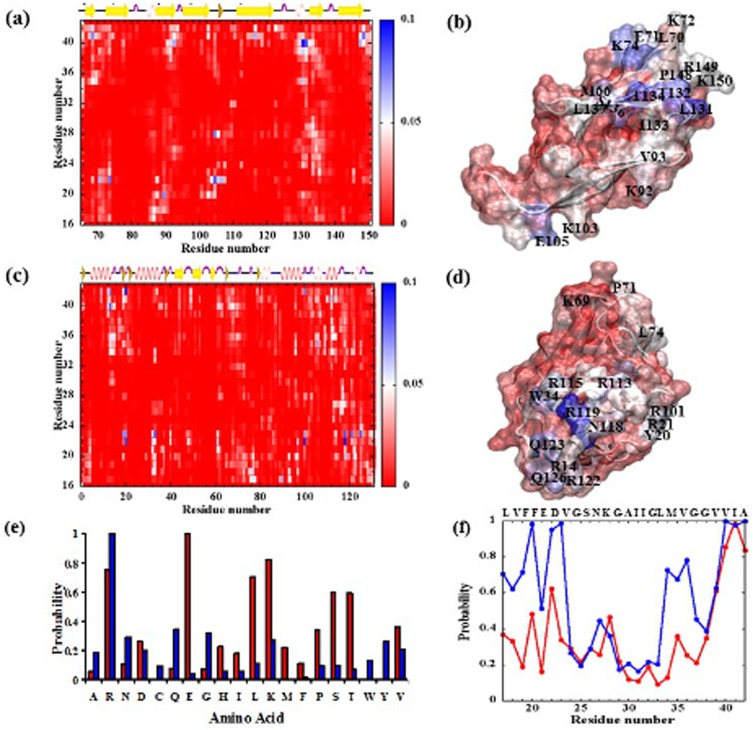
Probability of inhibitor-peptide contact formation. Ensemble-averaged pairwise contact maps (a) between ACD and Aβ and (c) between lysozyme and Aβ. Data shown is averaged over all ∼200 ns runs. X and Y axes show residues from the inhibitor protein and Aβ_17–42_, respectively. A contact between residue *i* from the inhibitor and residue *j* from Aβ is considered, if any heavy atom from residue *i* is within 5 Å of any heavy atom from residue *j*. The contacts are color-coded according to the color scale shown in right. The secondary structure assignment of the inhibitor is shown on top. The molecular surfaces of the inhibitor proteins ((b) ACD and (d) lysozyme) are also shown, which is color-coded red to blue (low to high) according to the probability of contact formation with Aβ. (e) Contact probabilities for each residue type of ACD (red) and lysozyme (blue). (d) Residue-based contact probabilities of the Aβ_17–42_ peptide with ACD (red) and with lysozyme (blue).

Why ACD-peptide binding is less extensive than the inter-peptide binding, whereas the opposite effect is observed for lysozyme? To answer this question, we compared the binding interactions of Aβ peptides with ACD and with lysozyme by estimating their ensemble-averaged relative contact probabilities (**Fig. 4** **)**. Two distinct regions of the crystallin domain are primarily found to interact with Aβ (**Fig. 4a-b**); (i) the β4–β8 pocket and (ii) the top β-sheet (β2, β3 and β9 strands). Previous studies suggest that these regions comprise many surface-exposed residues that are implicated in substrate binding [65]. The buried ACD dimer interface comprising the β6+7 strands lacks interaction with amyloid peptides, as expected (**Fig. 4a-b**). From the peptide side, both termini tend to interact with ACD. Only a few contacts are observed in the middle turn region (residues 24–29) except for K28. Consistently, the highly probable ACD-Aβ residue pairs include L131-V40, T132-V40, R149-I41, R74-E22, R107-E22, and E105-K28.

Similar analysis of the lysozyme-Aβ interaction shows that arginines from lysozyme that are dominate binding with the Aβ peptide (**Fig. 4c-d**). In fact, arginines are prevalent in lysozyme sequence (14 in total). Residues with high Aβ contacting probability include R14, R21, R101, R115, R119, and R122, which are all located within the alpha-domain (**Fig. 4d**). Some additional contacts with proximal hydrophobic (Y20, W34) and polar (Q123 and Q126) residues are also observed (**Fig. 4d**). Consistently, the highly probable contacts involve residues R21, R101, and R119 from lysozyme and residues E22, D23, and A42 (with negatively charged C-terminus) from Aβ.

The contact probability per amino acid type analysis (**Fig. 4e**) reveals that both positively and negatively charged residues (Arg, Lys, Glu) of ACD dominate Aβ-binding, while significant participation of the polar (Ser, Thr), and hydrophobic (Leu, Pro, Val) residues is also observed. These results imply that the ACD contact surface is more heterogeneous in terms of the amino acid composition, as no particular amino acid type shows a strong binding preference for Aβ. This result is consistent with the heterogeneous sequence distribution of the ACD surface (**Fig. 1a**). We further estimated the residue-specific contact probability of Aβ (**Fig. 4f**). L17, F20, E22, K28, and the carboxyl-terminated C-terminus (^39^VVIA^42^) from Aβ often contact ACD. The higher contact probability of the mainly hydrophobic C-terminus is consistent with their frequent interaction with the β4, β8, and β9 strands of ACD (**Fig. 4a**). Given the more heterogeneous nature of the ACD surface, it is likely difficult for the Aβ N-terminus (+NH_3_-^17^LVFFAED^23^) to find a complementary binding pocket that can satisfy all or majority of the possible charge-charge interactions. Thus, the C-terminus (with a smaller local concentration of charged residues) appears to remain in contact with ACD more frequently compared to the N-terminus. The more heterogeneous ACD surface also explains why a single Aβ monomer is less likely to form an extensive contact surface with ACD (in contrast to what observed with lysozyme, **Fig. 2** **–** **3**) and thus is not stable, which lead to ACD's preference for the peptide oligomers.

In contrast, arginines from lysozyme alone control the binding with Aβ (**Fig. 4e**). Much stronger participation from the Aβ N-terminus (mainly near the acidic residues) is noticed in lysozyme binding compared to that observed for ACD binding, suggesting that attractive electrostatic forces dominate lysozyme-Aβ interaction. The C-terminus and residues 34–38 also exhibit high contact probability. The high lysozyme-contacting probability of several Aβ residues is consistent with a more extensive contact surface (**Fig. 3a**). Overall, the arginines in the alpha-domain of lysozyme drives formation of a stable, extensive contact surface with the negatively charged residues of the Aβ peptides in their monomeric form, which is further accompanied by a few hydrophobic and polar contacts. Such extensive interaction allows lysozyme to compete more efficiently with the inter-peptide interaction compared with that for ACD.

Given the importance of the D23-K28 salt-bridge in maintaining the rigidity of the hairpin-like monomeric structure in the oligomers [66] and fibrils [8], [11], we further evaluate the interaction of those two residues (D23 and K28) with the two inhibitor proteins. Overall, K28 shows a ∼40% contact probability with ACD and contacts strongly with E105, E106, T134, and S135. D23 interacts with ACD with ∼30% probability, particularly with K74, T134, and N146. On the other hand, residues E102, N104, and N118 from lysozyme contact with K28 of Aβ that shows an overall contact probability of 40%. D23 of Aβ strongly contributes to lysozyme binding (with 100% probability) and contacts with W34, R119, R122, and Q126. In summary, both inhibitor proteins strongly engage residues D23 and K28 of Aβ *via* electrostatic interactions.

### ACD and lysozyme remain structurally intact upon Aβ binding

Next, we investigated if the inhibitors undergo any structural change upon Aβ binding or not. The evolutions of the root-mean-square distance (RMSD) from the native structure show that both ACD and lysozyme remain quite stable over the simulation time (**Fig. S5a** in File S1). Lysozyme appears relatively more stable with RMSD fluctuating around 1.5 Å. The RMSD of crystallin domain fluctuates between 2 and 4 Å. The root-mean-square-fluctuation (RMSF) per residue plot (**Fig. S5b** in File S1) demonstrates that all secondary structure elements of crystallin domains remain unperturbed upon Aβ interaction, while the loops connecting the strands fluctuate. These results are consistent with the reported high stability of the ACD dimer observed in chemical denaturation experiments [67] and also with the hyperthermophilic nature of this protein. For lyszoyme, only α5 and α7 helices exhibit RMSF values higher than 1.5 Å, while the rest of the secondary structural elements remain highly intact (**Fig. S5b** in File S1). Overall, the results imply that both inhibitors remain structurally unperturbed upon peptide binding.

### Effect of inhibitor binding on the Aβ conformation

**Figure 5:**
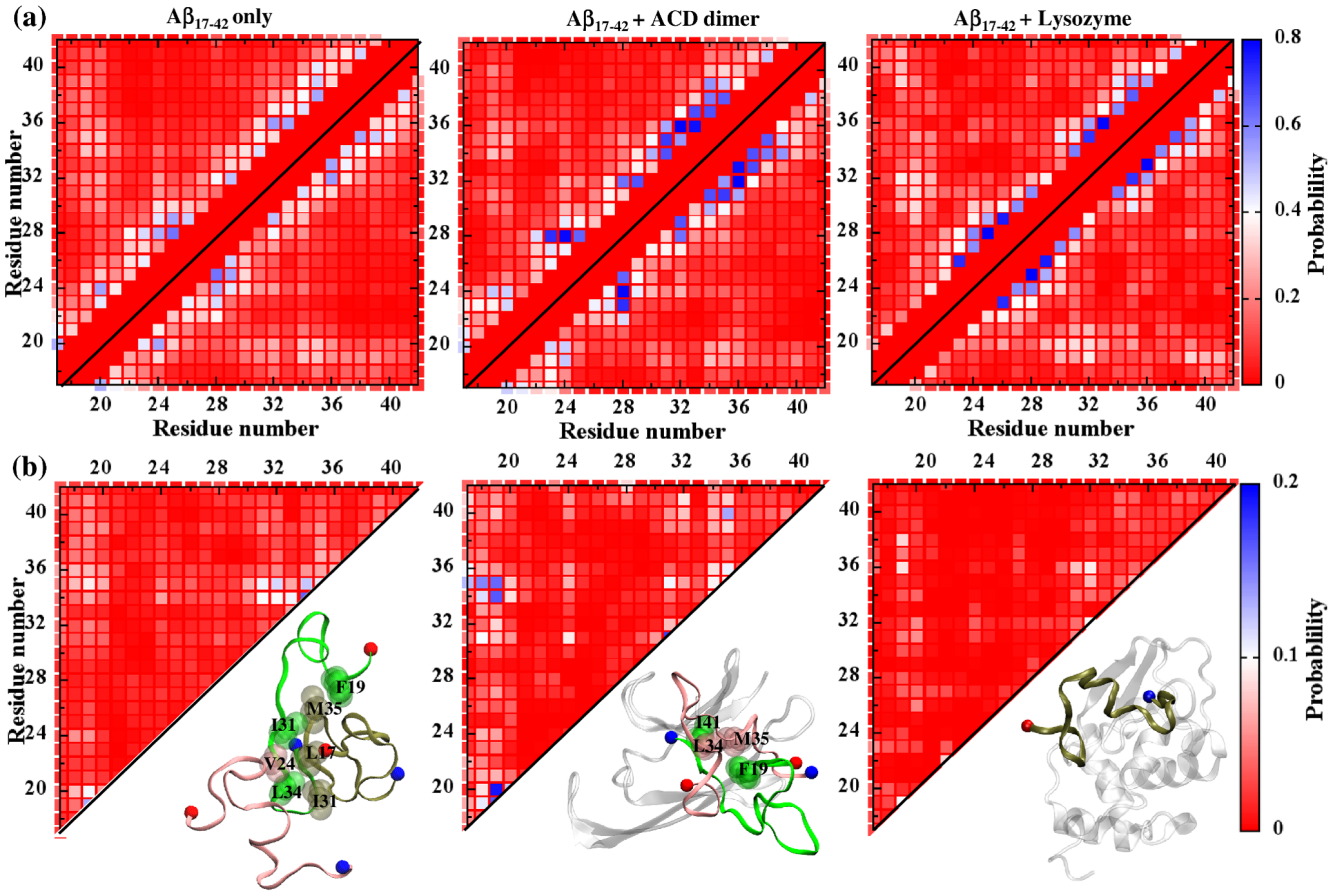
Probability of peptide-peptide contact formation. Ensemble-averaged probabilities of (a) tertiary contact and (b) quaternary contact formation of Aβ peptides in the control system (left), ACD-bound (middle), or lysozyme-bound (right) obtained from an ensemble generated from at least five ∼200 ns runs. The size of the ensemble is>10000 conformations. The peptide is considered complexed, if it forms more than five contacts with ACD/lysozyme. Only the non-sequential tertiary contacts (that are not formed between neighboring residues (*i+1*, and *i+2*) in sequence) are shown. The contacts are color-coded according to the color scale shown on the right. Snapshots of the oligomeric conformations with prevalent inter-monomer contacts are shown at bottom. Peptides are colored in green, pink, or tan, whereas ACD/lysozyme is colored in white. The N– and C-termini of the peptides are colored in red and blue, respectively. For the ACD-bound oligomer, only the inter-peptide contacts that form with higher probability compared to the free oligomer are shown for clarity.

We further analyzed the effect of inhibitor binding on the structure of Aβ peptides. For this purpose, an ensemble consisting of >10,000 peptide structures were used for each case. A peptide is considered to be complexed, if it forms more than five contacts with ACD/lysozyme. The secondary structure (**Fig. S6** in File S1) as well as the tertiary and quaternary contact probabilities **(** **Fig. 5** **)** were estimated. The ensemble-averaged secondary structure propensity (**Fig. S6a** in File S1) of the peptides in the control system reveals that coils and turns dominate at ∼33% and ∼55%, respectively, suggesting a mainly unstructured Aβ_17–42_. This finding is consistent with earlier replica-exchange simulations of Aβ_17–42_ [68]. Similar results are found for the complexed peptides, suggesting insignificant secondary structure change upon inhibitor binding. Some transient α-helix formation is noticed in the C-terminal region of Aβ upon ACD interaction (**Fig. S6b** in File S1).

The tertiary contact probability plot for the free peptides suggests substantial short-range interactions along the sequence (**Fig. 5a**). Residues 18–20 from CHC form weak, long-range, hydrophobic contacts with residues 30–40, especially with L34 and M35 (with a probability of ∼35%), similar to that reported for the hairpin-like conformation in the NMR-based fibril structure [9], [10]. Upon ACD binding, the tertiary structure of Aβ shows some changes. Short-range interactions, especially those near residues K28 and in the C-terminus (residues 32–38), become stronger upon ACD interaction, consistent with observed transient α-helix formation in this region (**Fig. S6b** in File S1). In the lysozyme-bound peptides, residues 19–21 show slightly higher contact probabilities with residues 29–32 and residues 39–41, when compared with the free peptides. In addition, lysozyme binding also induces local interaction within the peptide, but to a lesser extent to that seen for the ACD-bound peptide.

**Figure 5b** shows the quaternary contact probability plots for Aβ peptides. Results for the free peptides reveal that all regions except residues 21–23 and 25–29 are involved in the inter-peptide association, with residues 31–35 showing relatively higher tendency. These results are in overall good agreement with earlier experimental [69] and simulation results [70], suggesting the importance of these hydrophobic regions in inter-peptide interaction. Upon complexation with ACD, hydrophobic residues from the N-terminus (e.g. residues F19, and F20) and from the C-terminus (e.g. residues L34 and M35) show enhanced propensity to interact with other peptides. **Figure 5b** shows snapshots of these oligomers with hydrophobic contacts formed at the inter-monomer interface. In contrast, very few inter-peptide contacts appear in lysozyme-complexed peptides, suggesting that the extensive lysozyme-Aβ monomer attraction does not allow formation of the hydrophobic inter-monomer interface. Thus, the inter-peptide contact appears limited upon lysozyme binding.

## Discussion and Conclusion

Several studies have suggested that αB-crystallin can protect the cell from Aβ aggregation and toxicity at sub-stoichiometric ratios [31], [35], [36]. Single-molecule experiments performed at an equimolar ratio also suggested formation of stable complexes between αB-crystallin and small Aβ_1–40_ aggregates (n = 2–10) during disaggregation reaction [33]. Recently, a designed dimer consisting only the α-crystallin domain has been shown to act as a potent inhibitor of amyloid fibril formation and toxicity [25], which is very similar to our simulated system of ACD dimer. Separately, human lysozyme, which is not a chaperone, can completely inhibit Aβ aggregation at equimolar lysozyme: Aβ_1–40_ ratio [39]. The same study reported lag time delays of Aβ peptide aggregation at 1∶10 ratio of lysozyme and peptide. In line with these experiments, the current study shows that both lysozyme and the ACD dimer inhibit early oligomerization of Aβ_17–42_ peptides at 1∶10 ratio by competing with inter-peptide interaction. However, the simulations indicate that the mechanisms by which α-crystallin domain and lysozyme interact with Aβ_17–42_ are quite different. Aβ monomers bind with ACD *via* limited interactions due to the more heterogeneous nature of the ACD surface. The resulting complexes are transient. Nevertheless, small Aβ oligomers form more stable complexes with ACD, as the peptides can collectively form higher number of contacts with ACD. These ACD-bound peptides also demonstrate stronger inter-peptide hydrophobic contacts. Thus, the ACD-bound oligomers remain stable for ∼100 ns. Mainly charged residues as well as some hydrophobic and polar residues from the top β-strands and the β4-β8 pocket of ACD participate in Aβ-binding.

It should be mentioned that the flanking regions of ACD (the variable, largely unstructured N-terminal region and the moderately conserved C-terminal extension) [20] are not included in our simulations. It is likely that those flanking regions, particularly the 65 residue long N-terminal region, also contribute to Aβ binding. Several studies suggest various binding sites within αB-crystallin, which include both ACD and the flanking regions, but there is little agreement [24], [35], [71], [72]. In the present study, those flanking disordered regions are not included in the model to keep the computational expense reasonable. It should be also mentioned that, ACD alone, in both monomeric and dimeric forms, is sufficient for inhibiting Aβ_42_ aggregation and toxicity [25], which supports the relevance of our simulations in understanding the amyloid inhibitor activity of αB-crystallin.

In contrast, human lysozyme can form a large number of attractive electrostatic interactions due to the prevalent presence of locally concentrated arginines on its surface. Consequently, lysozyme can simultaneously bind with multiple Aβ monomers and form longer-lived complex with them. These results emphasize the importance of the surface composition of an inhibitor protein in determining the details of the molecular mechanism of amyloid inhibition. In addition, these simulations highlight the importance of the electrostatics in the Aβ-inhibitor interaction, consistent with recent experiments [38].

Interestingly, Aβ monomers have been reported to be neuro-protective [73], [74], whereas the small oligomers (n = 2–4) as well as dodecamers and protofibrils are associated with neurotoxicity [75]–[77], indicating a “loss of function” by pathological aggregation of Aβ peptides. In addition, small oligomers (n = 2–4) are efficient in nucleating assembly; trimer and tetramer being more efficient than the dimer [78]. Experiments show that the toxic Aβ_1–42_ oligomers maintained their toxicity in presence of hen egg white lysozyme that shares a very high structural (RMSD = 0.65 Å) and sequence similarity (61%) with human lysozyme; however, the toxicity was significantly reduced in presence of αB-crystallin [37]. More recent experiments indicate that human lysozyme suppresses Aβ_1–40_ aggregation and also to some extent toxicity at a 1∶1 molar ratio [79]. Taken together, previous experiments[30], [31], [36], [37], [39] suggest that natural chaperones that are of direct biological relevance, such as αB-crystallin, can exhibit an inhibitory effect on both Aβ aggregation and toxicity at greatly sub-stoichiometric concentrations. In contrast, human lysozyme, a non-chaperone protein, can inhibit Aβ aggregation and to some extent toxicity at much higher stoichiometric concentrations [79]. A non-specific binding between lysozyme and random coil Aβ monomers with some hydrophobic contribution was also suggested [79], which is overall in good agreement with our results. The findings of this study further imply that the molecular chaperone is naturally designed to inhibit amyloid aggregation by preferentially forming stable complexes with small oligomers (as opposed to a non-chaperone protein binding to monomers), which is determined by its heterogeneous surface composition. We hypothesize that the natural preference of the molecular chaperones for smaller amyloid oligomers may help optimizing their protective action. Overall, we present a dynamic molecular picture of the Aβ peptides interacting with two amyloid inhibitors by using atomistic simulations, which may serve to guide the rational design of more effective chaperones and amyloid inhibitors.

## Model and Method

The initial structure of a dimer of the α-crystallin domain (ACD) (**Fig. 1a**) was taken from the crystal structure deposited in the Protein Data Bank (residues 66–150 from PDB ID code 2wj7) [80]. The ACD forms an immunoglobin-like β-sandwich fold comprising strands β2–β9, and the assembly unit is a dimer (**Fig. 1a**). The initial coordinates of the monomeric Aβ_17–42_ was obtained from the NMR structure of the Aβ_1–42_ fibrils (PDB ID code 2beg). The protonation state of the peptide was set to that at pH 7 for all simulations. The ACD dimer was placed in the center of a ∼124 Å×124 Å×124 Å^3^ cubic box of water containing 100 mM NaCl to closely mimic physiological conditions. Ten Aβ_17–42_ peptides with charged termini were placed in random orientation at the center of the faces and at alternating vertices of the cubic box. This results in the simulated concentration of ∼8.7 mM Aβ that is much higher than what is typically used in experiments (∼10–50 µm) [30], [31], [36]. Thus, a much higher Aβ concentration compared to the experiments was used to expedite the association kinetics in simulations by reducing the time for random diffusion.

The minimum distance (only considering heavy atoms) between the ACD dimer and Aβ peptide was set to 15 Å. Ions were added to neutralize the net charge of the system (-2e for each ACD domain, -1e for Aβ_17–42_). **Figure 1d** illustrates one typical system of the solvated system with ten Aβ peptides and one ACD dimer. This system, consisting of a total of ∼180,000 atoms, was first energy-minimized for 50,000 steps followed by a 1 ns equilibration with a 0.5 fs time step.

At least five different MD runs were performed at 325 K and 1 atm for each of the three systems starting from the equilibrated structure: (*i*) the control system containing ten peptides (∼164,000 atoms in a 120×120×120 Å^3^ cubic box), (*ii*) ten peptides and one ACD dimer (∼180,000 atoms in a 124×124×124 Å^3^ cubic box), and (*iii*) ten peptides and one lysozyme molecule (∼164,000 atoms in a 120×120×120 Å^3^ cubic box). The system containing one lysozyme molecule and ten peptides was prepared following a similar protocol, in which one human lysozyme molecule (residues 1–130, PDB ID code 1rex, +8e charge) was placed in the center of the simulation box. Each simulation was started from different initial coordinates and velocities assigned from a Maxwell-Boltzmann distribution at the specified temperature. The initial orientation of the ACD dimer (or lysozyme) and the initial location of the peptides in the box were different in each run to remove bias of the initial structure on the final results. To rule out the possibility that the initial coordinates of the Aβ peptides taken from a fibril structure might affect our final conclusions, we plotted the evolutions of the C_α_-RMSD of all ten peptides from the initial structure during the first 10 ns of MD of the control system in **Fig. S7** in File S1. Within first 4–5 ns, the C_α_^∼^ RMSD of those peptides reaches a value of 6–10 Å, consistent with their disordered nature. This result confirms that our conclusions are independent of the choice of the Aβ initial coordinates. The simulations were performed for 200 ns or longer. The total aggregate simulation time was ∼4.5 µs.

The relative concentrations of ACD and Aβ are similar to that used in experiments, in which αB-crystallin are found to interact with Aβ variants and inhibit their aggregation and toxicity [30], [31], [36], [37]. Use of multiple peptides in the simulation system allows exploration of more than one binding sites on the inhibitor proteins as well as their interaction preference for peptide monomers vs. oligomers. It should be noted that exact size prediction of the inhibitor-complexed oligomers is beyond the scope of this study and thus the size reported here should be considered in a more qualitative manner. The exact size of the complexed oligomers can be affected by several factors such as the finite size of the system, simulation timescale, and the relative concentration of inhibitor and peptides.

The particle-mesh Ewald (PME) method was used for the long-range electrostatic interactions [81], while the van der Waals interactions were treated with a cut-off distance of 12 Å. The CHARMM22 [82] force field with CMAP extension [83] was used for the proteins. This force-field has been widely used to simulate both structured proteins [84], [85] and unstructured peptides [86]–[91]. For water, a modified TIP3P water model with its bond lengths constrained with SHAKE/RATTLE [92] was used. All simulations were performed using the NAMD2 [93] molecular modeling package using IBM Blue Gene supercomputers with a 2 fs time step in a NPT ensemble at 325 K and 1 atm. The temperature was controlled using the Langevin dynamics scheme, and the pressure was controlled using the Nosé-Hoover Langevin piston pressure control pressure coupling implemented in NAMD.

A cutoff distance of 5 Å between heavy atoms was considered to define a contact between two residues. A peptide was considered to be the component of an oligomer that is complexed with inhibitor, if it simultaneously forms more than five contacts with the inhibitor and another peptide. For the quaternary contact probability determination, all dimeric combinations of peptides, in which any one monomer forms more than five contacts with the inhibitor, were considered. The secondary structure was determined using the STRIDE program [94].

The residence times of the inhibitor-bound peptides were estimated by means of a survival time correlation function S(t), similar to what has been widely used to compute water residence time in simulations [95]. The correlation function can be defined as 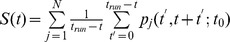; where *N* is the number of peptides in the system. The binary function 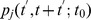 = 1, if the peptide labeled *j* remains inhibitor-bound (forms more than five heavy atom contacts with the inhibitor) from time *t* to to *t+t^'^*. *S(0)* gives the average number of complexed peptides and *S(t)* gives the average number of peptides that still remain bound after a time *t*. *t_0_* was taken to be 1 ns to allow for short excursion. Averages were calculated over all configurations sampled during a single trajectory. Results obtained from different runs were further averaged.

The convergence of the binding simulations was confirmed by several reversible binding events of the peptides to both ACD and lysozyme during ∼200 ns of MD (see **Fig. S8** in File S1). However, it should be noted that the ∼200 ns timescale might not be adequate for the full exploration of the structural changes of the peptide upon oligomerization and/or complexation. Approaches such as replica exchange, similar to previously performed work [96], therefore will likely be required, which is beyond the scope of the current study.

## Supporting Information

File S1 **Figures S1–S8.** Figure S1. Evolutions of different sized Aβ species of the control system containing only 10 peptides. Data was averaged over 50 time interval. A1: Aβ monomer; A2–5: small Aβ oligomer (n = 2–5); A6–10: larger Aβ oligomer (n>5). Data for all six runs are shown. Each run is represented by a different color. Figure S2. Evolutions of different sized Aβ species of the system containing 10 peptides and an ACD dimer. Data was averaged over 50 time interval. X =  uncomplexed ACD; XA1: ACD-bound Aβ monomer; XA2–5: ACD-bound small Aβ oligomer (n = 2–5); XA6–10: ACD-bound larger Aβ oligomer (n>5). Data for all seven runs are shown. Each run is represented by a different color. Figure S3. Evolutions of different sized Aβ species of the system containing 10 peptides and human lysozyme. Data was averaged over 50 time interval. X =  uncomplexed lysozyme; XA1: lysozyme-bound Aβ monomer; XA2–5: lysozyme-bound small Aβ oligomer (n = 2–5); XA6–10: lysozyme-bound larger Aβ oligomer (n>5). Data for all five runs are shown. Each run is represented by a different color. Figure S4. Evolutions of different ACD/lysozyme-complexed Aβ species. 1 ns running average and snapshots at ∼200 ns of aggregates in presence of ACD dimer (a) and in presence of lysozyme (b) are shown. Species An represents an Aβ aggregate of size n. Figure S5. Structural changes of amyloid inhibitor proteins. (a) Evolutions of C_α_ root-mean-square deviation (RMSD, in Å) from the native structure of ACD domains (left and middle panels) and of lysozyme (right panel). Different colors represent different runs. (b) Time-averaged root-mean-square fluctuation (RMSF, in Å) per residue from the native structure for the ACD domains and for lysozyme. Figure S6. Secondary structure analysis. (a) Total secondary structure population (in %) averaged over all ∼200 ns runs: black – control system; red – ACD-bound; green - lysozyme-bound. The standard deviations were estimated by splitting the data into two equal sets. (b) Residue-based α-helix and β-strand population (in %) averaged over all ∼200 ns runs. Some transient helix formation is notice for all three systems. ACD-bound peptides show slightly higher helix population in the C-terminal region. Figure S7. Structural changes of Aβ peptides. (a) Evolutions of the C_α_ root-mean-square deviation (RMSD, in Å) from the initial fibril structure of the ten Aβ peptides during first 10 ns of a particular control run. Each color corresponds to a different peptide. (b) The structures of the peptides (shown in ribbon representation) at 5 ns. Figure S8. Evolution of inhibitor-peptide contacts. Number of ACD-Aβ contacts (a) and lysozyme-Aβ contacts (b) during a typical run as a function of simulation time. Each monomer is shows using a different color. Several binding/unbinding events are observed during ∼200 ns simulation.
(DOC)
