# Supplementary material for: Interaction of Amyloid Inhibitor Proteins with Amyloid Beta Peptides: Insight from Molecular Dynamics Simulations
Source: PLoS One. 2014 Nov 25;9(11):e113041. doi: 10.1371/journal.pone.0113041 (PMC4244084; doi:10.1371/journal.pone.0113041)

**Supporting Information for Publication**

**Title: Interaction of Amyloid Inhibitor Proteins with Amyloid Beta Peptides: Insight from Molecular Dynamics Simulations**

**Authors: Payel Das^1*^, Seung-gu Kang^1^, Sally Temple^2^, Georges Belfort^3^**

**^1^ Computational Biology Center, IBM Thomas J. Watson Research Center, 1101 Kitchawan Road, Yorktown Heights, New York 10598, United States**

**^2^ Neural Stem Cell Institute, Rensselaer, New York 12144, United States**

**^3^ Howard P. Isermann Department of Chemical and Biological Engineering and Center for Biotechnology and Interdisciplinary Studies, Rensselaer Polytechnic Institute, Troy, New York 12180, United States**


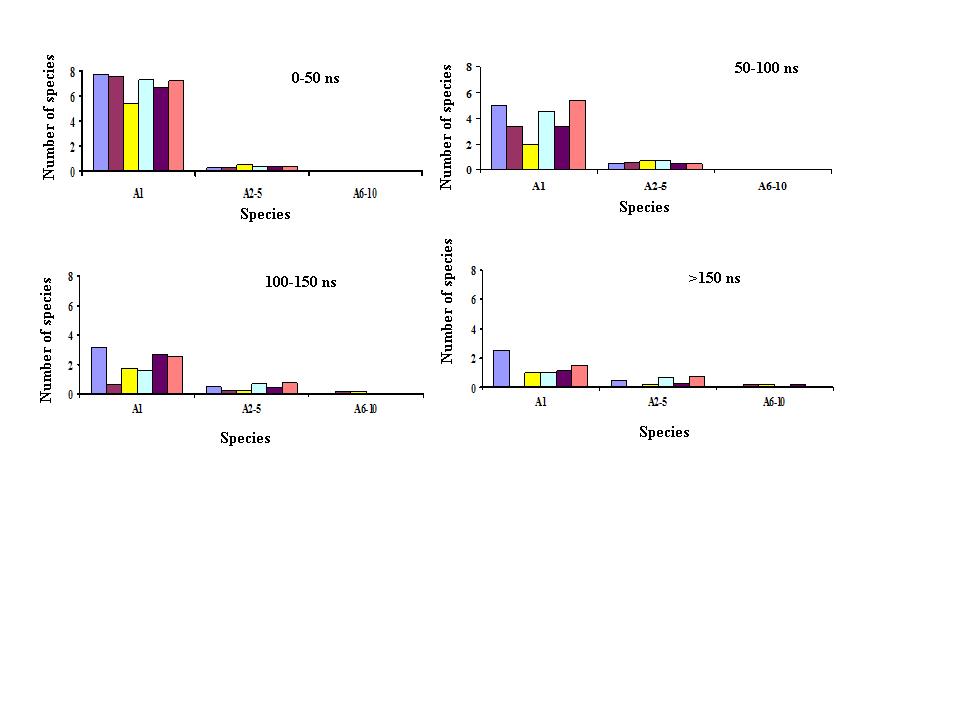
**Figure S1.** **Evolutions of different sized Aβ species of the control system containing only 10 peptides.** Data was averaged over 50 time interval. A1: Aβ monomer; A2-5: small Aβ oligomer (n=2-5); A6-10: larger Aβ oligomer (n>5). Data for all six runs are shown. Each run is represented by a different color.


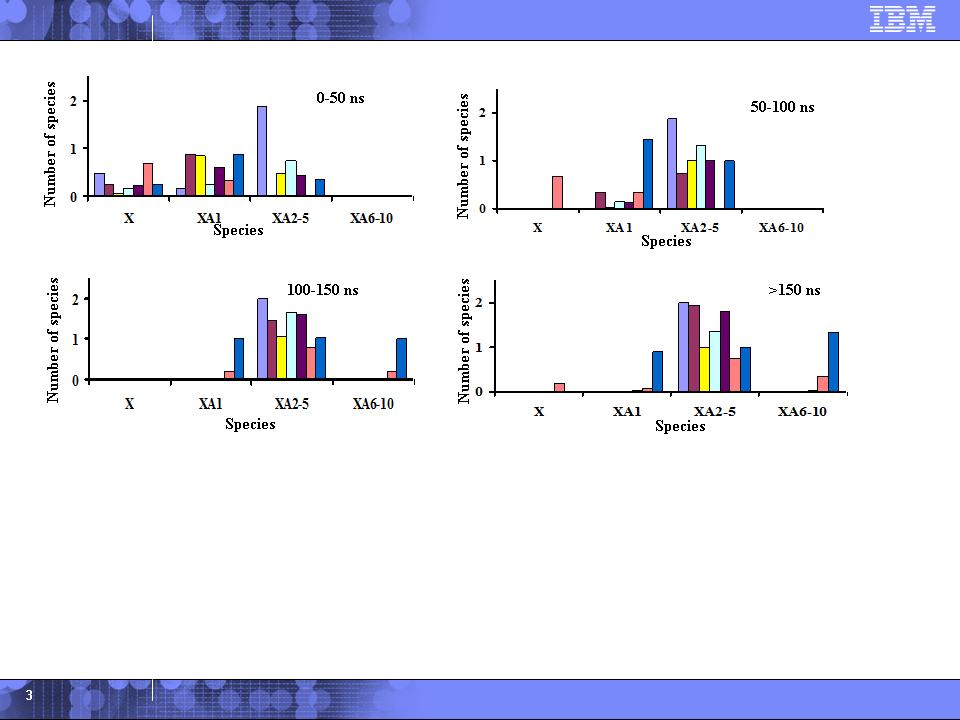
**Figure S2.** **Evolutions of different sized Aβ species of the system containing 10 peptides and an ACD dimer.** Data was averaged over 50 time interval. X= uncomplexed ACD; XA1: ACD-bound Aβ monomer; XA2-5: ACD-bound small Aβ oligomer (n=2-5); XA6-10: ACD-bound larger Aβ oligomer (n>5). Data for all seven runs are shown. Each run is represented by a different color.

**Figure S3.** **Evolutions of different sized Aβ species of the system containing 10 peptides and human lysozyme.** Data was averaged over 50 time interval. X= uncomplexed lysozyme; XA1: lysozyme-bound Aβ monomer; XA2-5: lysozyme-bound small Aβ oligomer (n=2-5); XA6-10: lysozyme-bound larger Aβ oligomer (n>5). Data for all five runs are shown. Each run is represented by a different color.


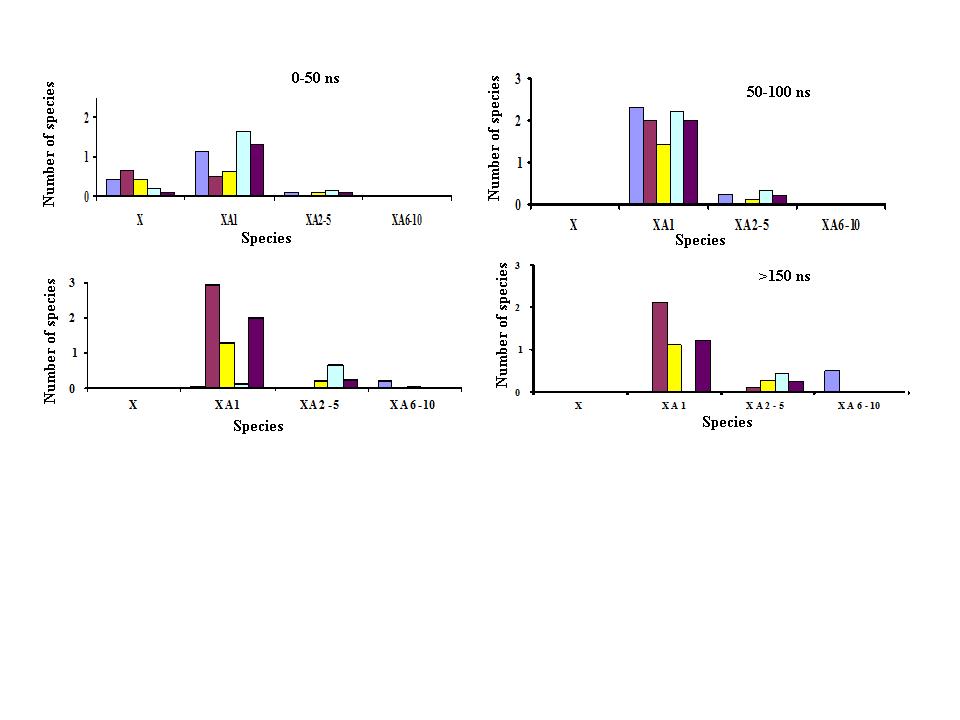


**Figure S4.** **Evolutions of different ACD/lysozyme-complexed Aβ species.** 1 ns running average and snapshots at ~200 ns of aggregates in presence of ACD dimer (a) and in presence of lysozyme (b) are shown. Species An represents an Aβ aggregate of size n.

**
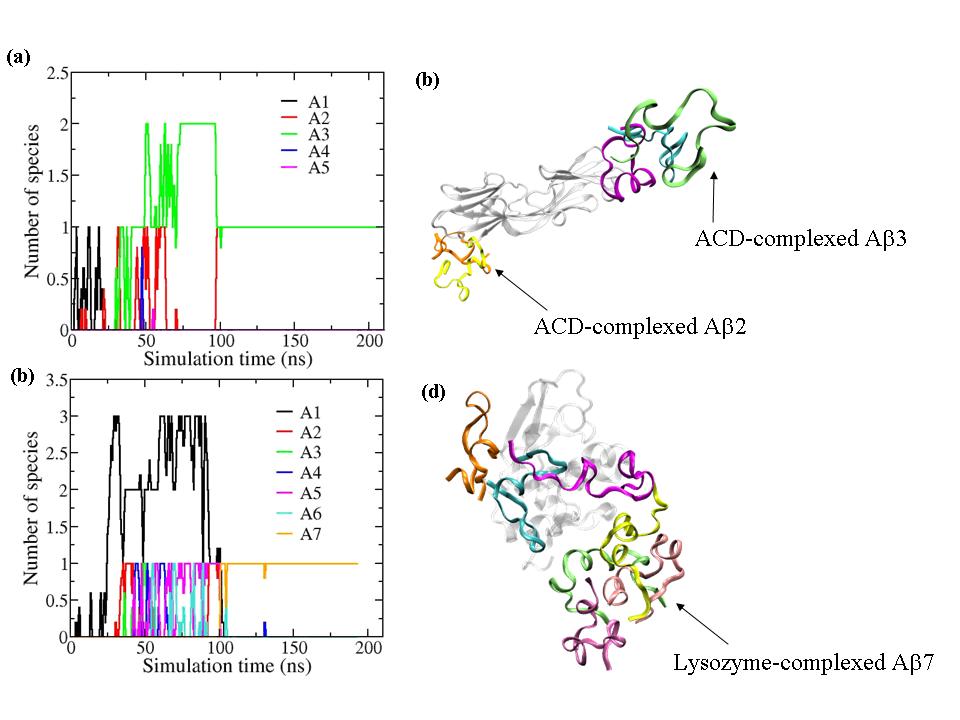
**

**Figure S5. Structural changes of amyloid inhibitor proteins.** (a) Evolutions of C_α_ root-mean-square deviation (RMSD, in Å) from the native structure of ACD domains (left and middle panels) and of lysozyme (right panel). Different colors represent different runs. (b) Time-averaged root-mean-square fluctuation (RMSF, in Å) per residue from the native structure for the ACD domains and for lysozyme.

**
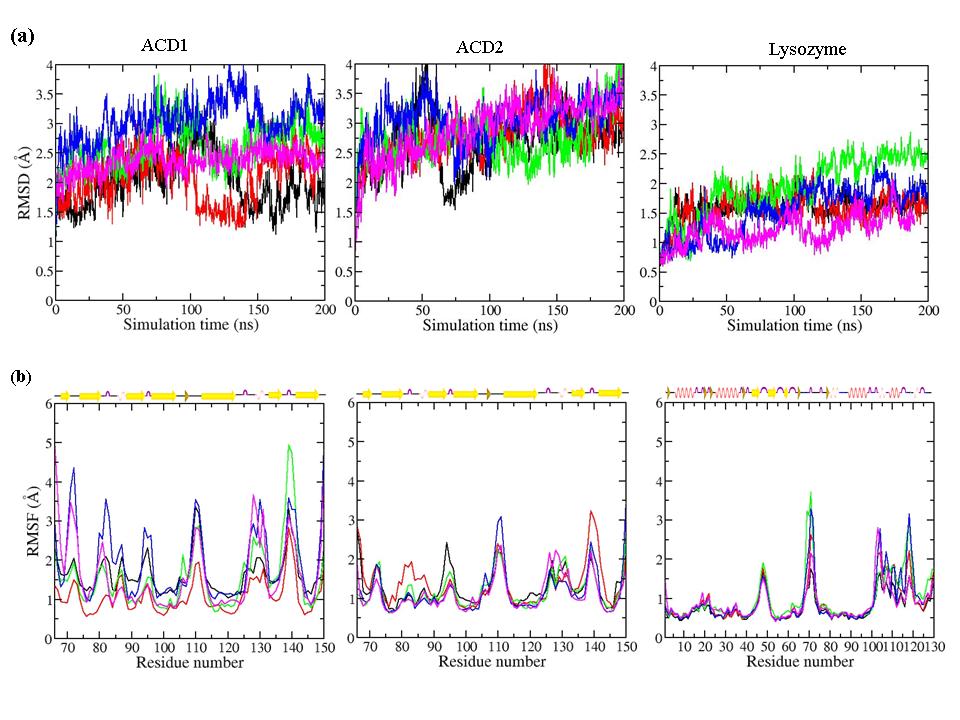
**

**Figure S6. Secondary structure analysis.** (a) Total secondary structure population (in %) averaged over all ~200 ns runs: black – control system; red – ACD-bound; green - lysozyme-bound. The standard deviations were estimated by splitting the data into two equal sets. (b) Residue-based α-helix and β-strand population (in %) averaged over all ~200 ns runs. Some transient helix formation is notice for all three systems. ACD-bound peptides show slightly higher helix population in the C-terminal region.

**
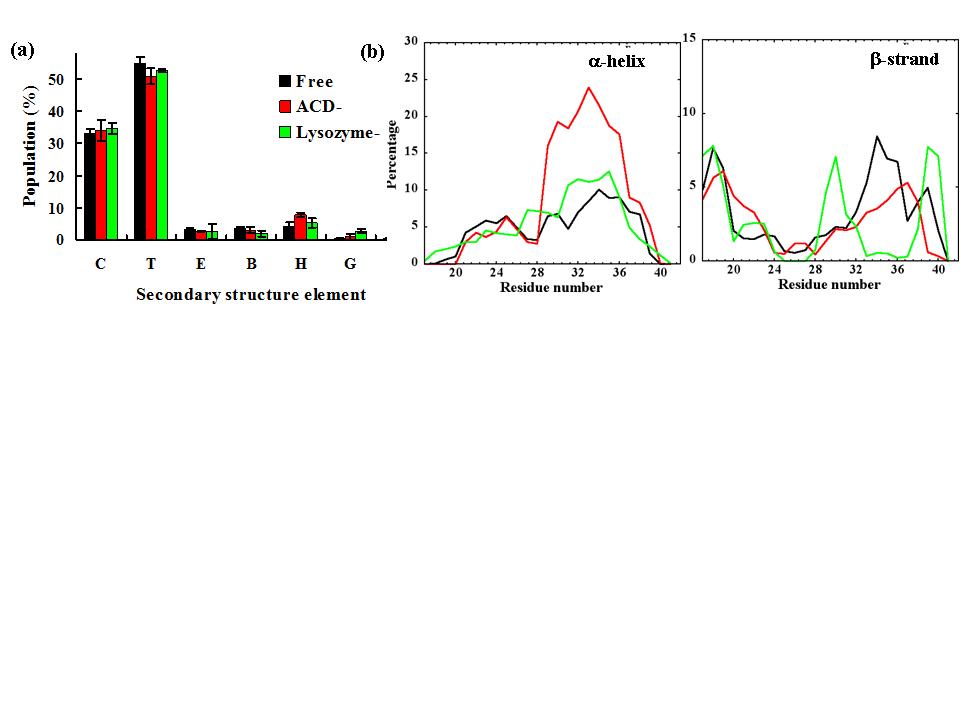
**

**Figure S7. Structural changes of Aβ peptides. (a)** Evolutions of the C_α_ root-mean-square deviation (RMSD, in Å) from the initial fibril structure of the ten Aβ peptides during first 10 ns of a particular control run. Each color corresponds to a different peptide. **(b)** The structures of the peptides (shown in ribbon representation) at 5 ns.


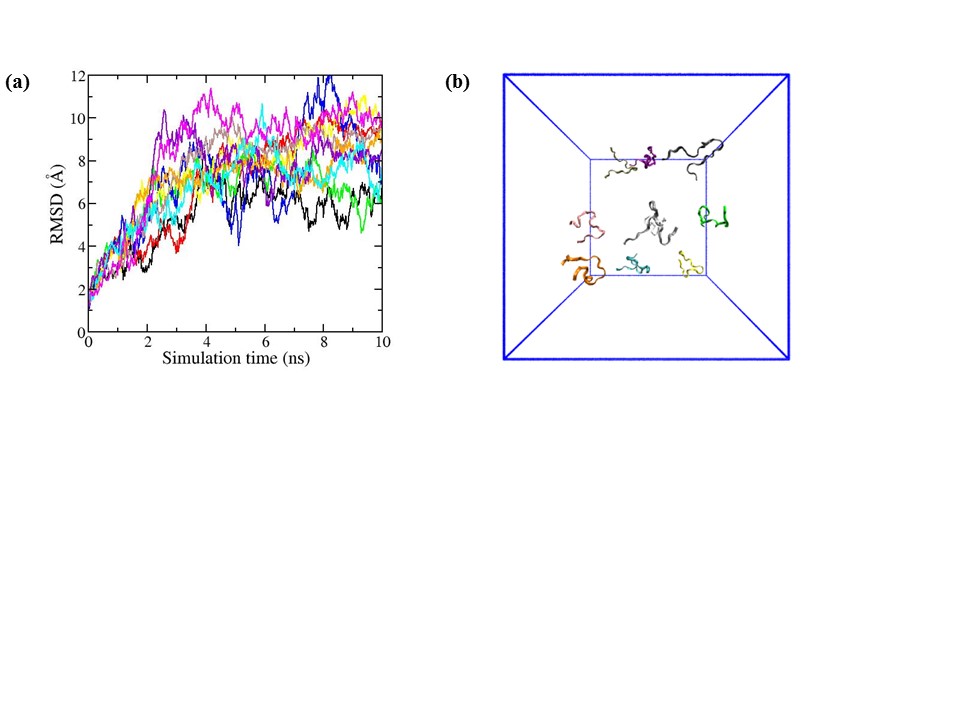


**Figure S8.** **Evolution of inhibitor-peptide contacts.** Number of ACD-Aβ contacts (a) and lysozyme-Aβ contacts (b) during a typical run as a function of simulation time. Each monomer is shows using a different color. Several binding/unbinding events are observed during ~200 ns simulation.
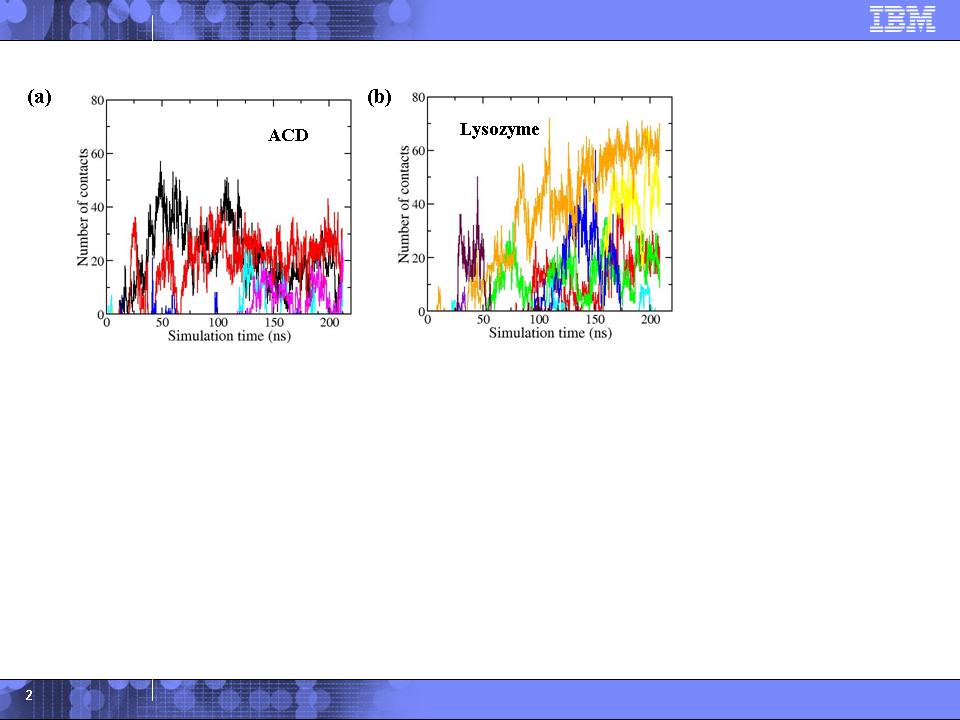

Supplement: File S1 — Figures S1–S8. Figure S1. Evolutions of different sized Aβ species of the control system containing only 10 peptides. Data was averaged over 50 time interval. A1: Aβ monomer; A2–5: small Aβ oligomer (n = 2–5); A6–10: larger Aβ oligomer (n>5). Data for all six runs are shown. Each run is represented by a different color. Figure S2. Evolutions of different sized Aβ species of the system containing 10 peptides and an ACD dimer. Data was averaged over 50 time interval. X = uncomplexed ACD; XA1: ACD-bound Aβ monomer; XA2–5: ACD-bound small Aβ oligomer (n = 2–5); XA6–10: ACD-bound larger Aβ oligomer (n>5). Data for all seven runs are shown. Each run is represented by a different color. Figure S3. Evolutions of different sized Aβ species of the system containing 10 peptides and human lysozyme. Data was averaged over 50 time interval. X = uncomplexed lysozyme; XA1: lysozyme-bound Aβ monomer; XA2–5: lysozyme-bound small Aβ oligomer (n = 2–5); XA6–10: lysozyme-bound larger Aβ oligomer (n>5). Data for all five runs are shown. Each run is represented by a different color. Figure S4. Evolutions of different ACD/lysozyme-complexed Aβ species. 1 ns running average and snapshots at ∼200 ns of aggregates in presence of ACD dimer (a) and in presence of lysozyme (b) are shown. Species An represents an Aβ aggregate of size n. Figure S5. Structural changes of amyloid inhibitor proteins. (a) Evolutions of Cα root-mean-square deviation (RMSD, in Å) from the native structure of ACD domains (left and middle panels) and of lysozyme (right panel). Different colors represent different runs. (b) Time-averaged root-mean-square fluctuation (RMSF, in Å) per residue from the native structure for the ACD domains and for lysozyme. Figure S6. Secondary structure analysis. (a) Total secondary structure population (in %) averaged over all ∼200 ns runs: black – control system; red – ACD-bound; green - lysozyme-bound. The standard deviations were estimated by splitting the data into two equal sets. (b) Re [file pone.0113041.s001.doc]
